# Machine or Human? Evaluating the Quality of a Language Translation Mobile App for Diabetes Education Material

**DOI:** 10.2196/diabetes.7446

**Published:** 2017-06-29

**Authors:** Xuewei Chen, Sandra Acosta, Adam E Barry

**Affiliations:** 1 Transdisciplinary Center for Health Equity Research Department of Health and Kinesiology Texas A&M University College Station, TX United States; 2 Texas A&M University Department of Educational Psychology Texas A&M University College Station, TX United States; 3 Texas A&M University Department of Health and Kinesiology Texas A&M University College Station, TX United States

**Keywords:** health literacy, health education, health communication, language translation, diabetes, machine translation, mobile translation app, human interpreter, translator

## Abstract

**Background:**

Diabetes is a major health crisis for Hispanics and Asian Americans. Moreover, Spanish and Chinese speakers are more likely to have limited English proficiency in the United States. One potential tool for facilitating language communication between diabetes patients and health care providers is technology, specifically mobile phones.

**Objective:**

Previous studies have assessed machine translation quality using only writing inputs. To bridge such a research gap, we conducted a pilot study to evaluate the quality of a mobile language translation app (iTranslate) with a voice recognition feature for translating diabetes patient education material.

**Methods:**

The pamphlet, “You are the heart of your family…take care of it,” is a health education sheet for diabetes patients that outlines three recommended questions for patients to ask their clinicians. Two professional translators translated the original English sentences into Spanish and Chinese. We recruited six certified medical translators (three Spanish and three Chinese) to conduct blinded evaluations of the following versions: (1) sentences interpreted by iTranslate, and (2) sentences interpreted by the professional human translators. Evaluators rated the sentences (ranging from 1-5) on four scales: Fluency, Adequacy, Meaning, and Severity. We performed descriptive analyses to examine the differences between these two versions.

**Results:**

Cronbach alpha values exhibited high degrees of agreement on the rating outcomes of both evaluator groups: .920 for the Spanish raters and .971 for the Chinese raters. The readability scores generated using MS Word’s Flesch-Kincaid Grade Level for these sentences were 0.0, 1.0, and 7.1. We found iTranslate generally provided translation accuracy comparable to human translators on simple sentences. However, iTranslate made more errors when translating difficult sentences.

**Conclusions:**

Although the evidence from our study supports iTranslate’s potential for supplementing professional human translators, further evidence is needed. For this reason, mobile language translation apps should be used with caution.

## Introduction

Diabetes is a major health crisis for Hispanics and Asian Americans. According to the Centers for Disease Control and Prevention (CDC), 29.1 million people (9.3% of the US population) have diabetes; 12.8% Hispanics and 9% Asian Americans above 20 years old were diagnosed with diabetes, compared to 7.6% non-Hispanic whites [1]. From 1997-2014, diabetes rates increased 103% for Asian Americans and 60% for Hispanics [2].

Compared to other ethnic groups, Hispanics and Chinese Americans are also more likely to have low English proficiency. Over 21% of the US population speaks a language other than English at home. Further, the highest percentages of individuals who speak no English are Hispanics and Chinese Americans [3]. Approximately 43.7% Hispanics and 55.7% Chinese Americans speak English less than “very well” [3] and would be considered having limited English proficiency (LEP). LEP refers to any person age 5 or older who self-reported speaking English less than “very well” [3]. In brief, because Hispanic and Chinese Americans are more likely to have LEP, communication challenges arising from language barriers might impact the quality of the health services and information they receive.

Populations with LEP encounter numerous health communication challenges due to barriers related to language proficiency. These language barriers, as many studies have pointed out, might lead to health disparities and poor health outcomes. For instance, individuals with LEP are more likely to take inaccurate medication dosages [4], have poor health status [5], spend additional money and time utilizing health care services [6], experience unsatisfactory events with health care providers, make improper health choices [7], and have limited access and use of preventive health care services [8]. For diabetes patients who have LEP, negative health outcomes include poor glycemic control [9] and diabetic retinopathy [10].

One potential tool for facilitating language communication between patients and health care providers is technology, specifically mobile phones. In the United States, smartphone ownership increased from 35% of the population in 2011 to 72% in 2016 [11]. These smartphone owners can access various apps including machine language translation apps. For instance, iTranslate is a mobile app available for mobile phones with Apple, Android, and Windows systems that instantly translates text or voice inputs and converts them into text and voice outputs. Such voice recognition features were developed from computerized systems.

There are no significant differences in smartphone ownership among different racial/ethnic groups [12]. Further, about three-quarters (73%) of the Hispanic smartphone owners have used their phones to search for health-related information, compared to 58% white and 67% black [13]. Smartphones with machine translation apps are efficient tools for helping populations with LEP overcome language barriers [14,15]. For instance, translation mobile apps might improve their understanding of health information and access to health resources.

However, translation inaccuracy has the potential to adversely impact information’s meaning and lead to negative health consequences. For example, language translation errors lead to misunderstandings about medical prescriptions [16] as well as misdiagnoses and mistreatments [17,18].

Previous studies have examined the usability of mobile language translation apps among clinicians and patients. In a study conducted by Abreu and Adriatico [19], the researchers investigated the experience of using the Google Translation App among a group of US audiologists and Spanish speaking patients/parents/guardians when they were communicating with each other. Abreu and Adriatico reported positive reactions from both the audiologists and the Spanish-speaking clients. Based on their findings, the authors concluded that the Google Translation App might be a viable tool for addressing language barriers and improving health communication when human interpreters were not available [19]. Similarly, Albrecht et al [20] examined the usage experience of a mobile translation app (xprompt) among nursing staff in Germany. The authors found that the participants perceived the xprompt app as useful for basic communication with non-German speaking patients [20]. Here, machine translation refers to automated computer translations powered by algorithms.

Besides usability, accuracy is another important criterion for evaluating machine language translation tools. With regard to the machine translation accuracy, previous studies assessed the translation product provided by Babel Fish and Google Translate websites using only writing inputs [21-24]. They noted that machine translation tools made errors when translating medical information [21-24]. Khanna et al [22] suggested that machine translation tools with a voice recognition feature might increase translation errors. Given the absence of research on voice recognition features and translations errors, we investigated the quality of a machine language translation mobile app with a voice recognition feature (iTranslate). Because diabetes is a major health crisis for Hispanics and Asian Americans [2], we selected diabetes patient education material. To the best of our knowledge, no study to date has investigated the quality of a mobile translation app interpreting spoken sentences.

The purpose of this pilot study is to evaluate the quality of iTranslate when interpreting spoken sentences from English to Spanish and Chinese. Our overarching research question is: Can iTranslate be an accurate and practical translation tool for patients-clinicians using diabetes education materials? To provide insights into this question, we posed the following research questions:

1. What is the quality of iTranslate when interpreting spoken sentences from English to Spanish, as compared to professional human interpreters?
2. What is the quality of iTranslate when interpreting spoken sentences from English to Chinese, as compared to professional human translators?

## Methods

### Materials to be Translated

We chose a publicly available diabetes patient education pamphlet as a heuristic example for this pilot study. The pamphlet, “You are the heart of your family…take care of it” (see Multimedia Appendix 1), is published by the National Institutes of Health and the CDC and distributed by the National Diabetes Education Program. This pamphlet contains two parts: Part A, six written sentences as behavior change suggestions for managing diabetes and Part B, three recommended questions for patients to ask their clinicians. Our study examined the quality of iTranslate when translating Part B. The study and results of Part A were reported by Chen et al [21].

### Procedures

**Figure 1:**
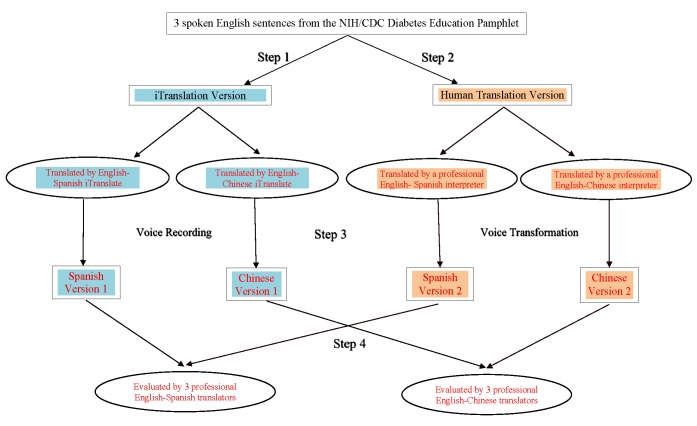
Four-step procedures.

This study was approved by the appropriate institutional review board. Figure 1 outlines the procedures employed throughout this study, which comprise four steps: Step 1. iTranslate mobile app translation process; Step 2. Human translation process; Step 3. Voice transformation; Step 4. Evaluation.

#### Step 1. Mobile Language Translation App

We used iTranslate app to translate three spoken questions from English into both Spanish and Chinese (Mandarin). We recorded these voice outputs into audio files.

#### Step 2. Human Translator

Two professional medical interpreters translated the three original English questions into Spanish and Chinese respectively. Both were American Translators Association (ATA) certified translators (one certified in English to Spanish and the other in English to Chinese). The ATA website lists all the certified translators’ contact information. We approached the translators as regular customers seeking and paying for translation services. We did not inform them that their translations would be evaluated. We emailed the original English questions to the translators, and they returned the translated sentences in audio files by email as well. We also asked them to provide transcriptions of their voice translations in a separate MS Word file.

#### Step 3. Voice Transformation

Since the evaluators might distinguish the machine from the human translation because of the potentially recognizable characteristics of the mechanical voice, we converted all the human voice translations into a machine voice. First, we copied and pasted the transcription on a voice transformation website and clicked the voice button for the three questions translated by the human interpreters to be converted to the machine voice function. Second, we reviewed the transcriptions and compared the machine voice to the original human voice (translators’ audio files) to ensure equivalency. Third, we recorded the three sentences, now in the machine voice, into audio files, and emailed these audio files to the evaluators. Table 1 lists the original English sentences and the translated Spanish and Chinese transcriptions.

**Table 1:** The original English and translated Chinese and Spanish versions of the sentences.

Original	iTranslate		Human	
English	Spanish	Chinese	Spanish	Chinese
What are my blood sugar, blood pressure, and cholesterol numbers?	¿Cuáles son mis azúcar en la sangre, presión arterial y colesterol?	我的血糖、血压和胆固醇是什么？	¿Cuáles son mis números de azúcar en la sangre, presión arterial y colesterol?	我的血糖、血压和胆固醇的值是多少？
What should they be?	¿Qué deberían ser?	他们应该是什么？	¿Cuáles deben de ser mis números?	正常值应该是多少？
What actions should I take to reach these goals?	¿Qué medidas debo tomar para alcanzar estas metas?	应该采取何种行动来达到这些目标？	¿Qué debo de hacer para alcanzar esas metas?	我应该怎么做来达到正常值？

#### Step 4. Evaluation

We sent invitation emails to the first 12 English-Spanish translators and 12 English-Chinese ATA certified translators listed on the ATA website. We emailed the survey package to the first six translators (three Spanish and three Chinese respectively) who accepted our study invitation. We asked them to evaluate the two versions of the voice translations (one by iTranslate app and the other one by the professional translator). Each evaluator received a US $15 check after submitting the evaluation survey package via email. The two interpreters who provided the human translation versions did not serve as evaluators, nor were they aware that their translations would be evaluated by other translators.

### Survey Package

To minimize rater bias and blind the evaluation process, the audio files were marked as version 1 (sentences translated by iTranslate) and version 2 (sentences translated by a human). The survey package contained one evaluation rubric in an MS Word file and two audio files (versions 1 and 2). We asked the evaluators to score each of the translated sentences using the evaluation rubric (see Table 2).

### Evaluation Rubric

We adapted the evaluation rubric from Khana et al [22], instructed the raters to evaluate the translated sentences based on four criteria—Fluency, Adequacy, Meaning, and Severity—on a 5-point scale (1 indicates the lowest quality and 5 indicates the highest quality). The Fluency and Adequacy criteria are standard domains for assessing machine translation quality [25]. Fluency assesses readability, grammar, and understandability. Adequacy assesses the amount of original information preserved. Meaning assesses the equivalency of the translation and the original sentence and detects misleading information [26]. Severity assesses the degree of the negative impact on a patient’s health outcome. Table 2 presents the four criteria and the description for each criterion.

**Table 2:** Rubric for evaluating translation quality.

	Fluency	Adequacy	Meaning	Severity
1	No fluency; no appreciable grammar, not understandable	0% of information conveyed from the original	Totally different meaning from the original	Dangerous to patient
2	Marginal fluency; several grammatical errors	25% of information conveyed from the original	Misleading information added/omitted compared to the original	Impairs care in some way
3	Good fluency; several grammatical errors, understandable	50% of information conveyed from the original	Partially the same meaning as the original	Delays necessary care
4	Excellent fluency; few grammatical errors	75% of information conveyed from the original	Almost the same meaning as the original	Unclear effect on patient care
5	Perfect fluency; like reading a newspaper	100% of information conveyed from the original	Same meaning as the original	No effect on patient care

### Data Analysis

**Figure 2:**
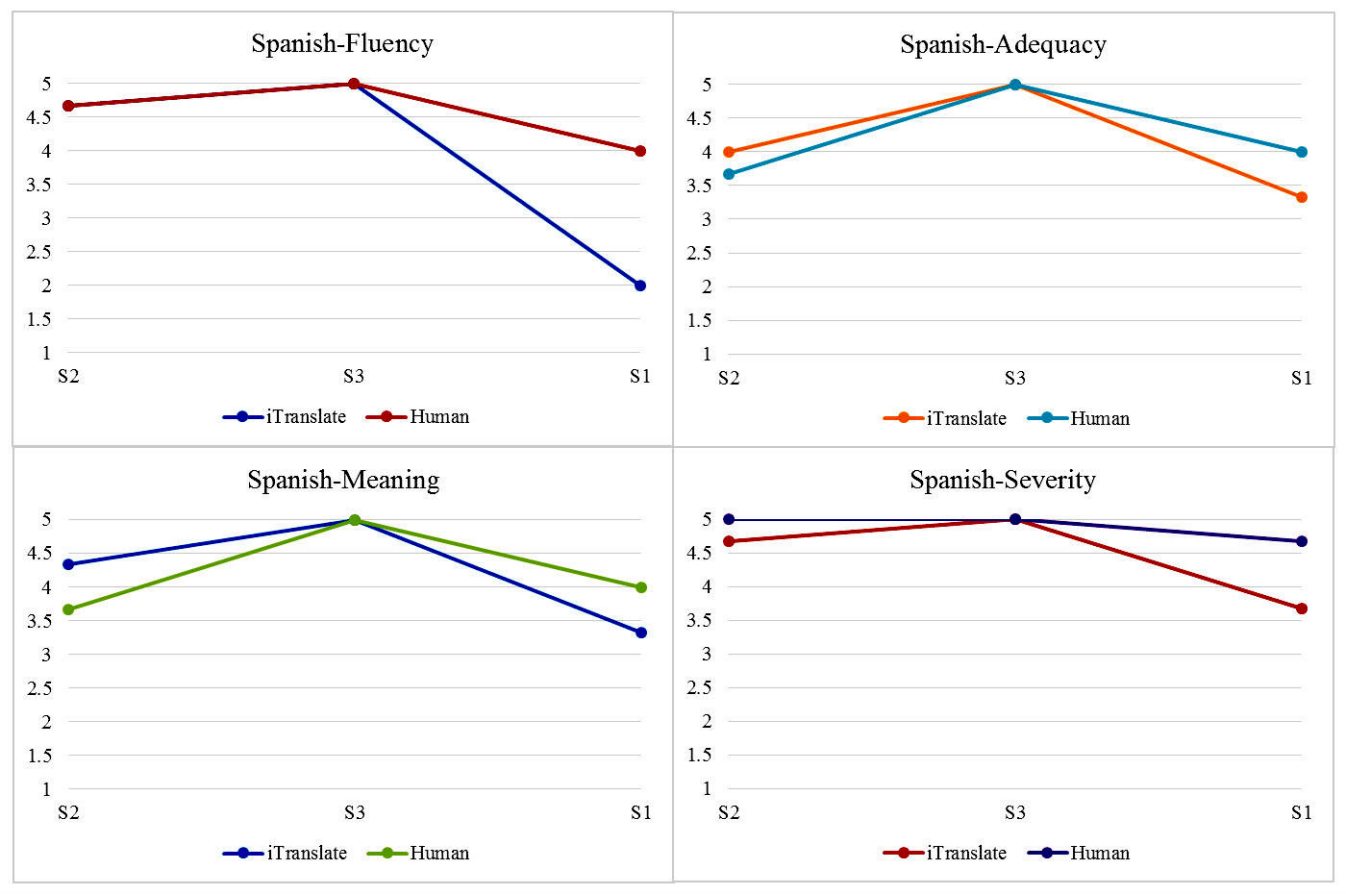
Scatterplots comparing Spanish iTranslate with the human translator scores.

**Figure 3:**
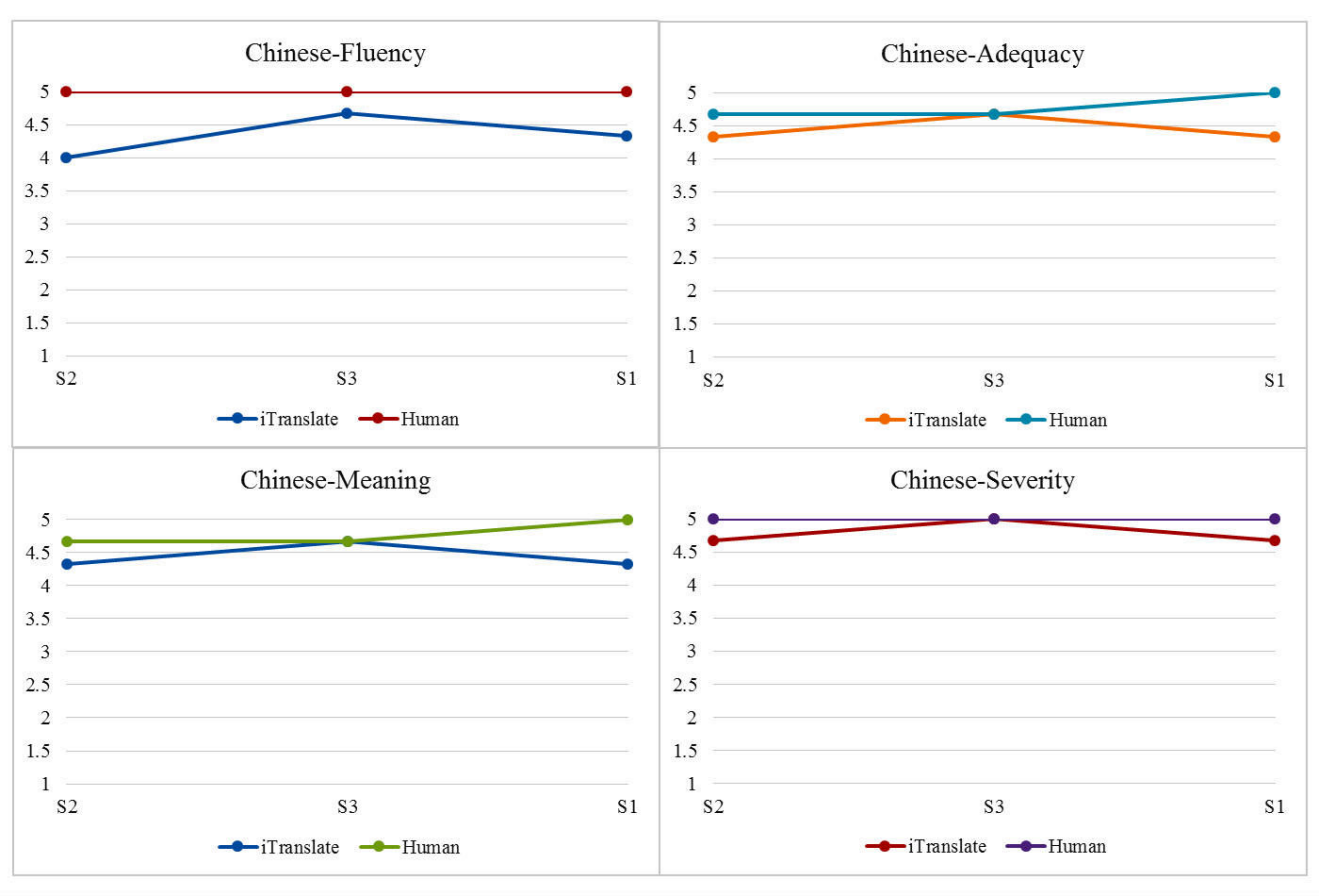
Scatterplots comparing Chinese iTranslate with the human translator scores.

We performed the Cronbach alpha test to assess the degree of rater agreement. Two sets of mean scores were calculated for each of the four domains (Fluency, Adequacy, Meaning, and Severity) on each sentence from the Chinese and Spanish rater groups. We also presented the readability statistics for each original English sentences. Readability statistics were generated using MS Word’s Flesch-Kincaid Grade Level, which assesses the degree of difficulty for readers to understand a sentence or paragraph [27]. For ease of comparison, two sets of graphs shown in Figures 2 and 3 visually depict the translation quality between iTranslate app and the human interpreters starting from the easiest to the most difficult sentence based on the readability statistics.

## Results

### Interrater Reliability

Cronbach alpha was used to assess the rating reliability across each evaluator. The Cronbach alpha values exhibited high degrees of agreement on the rating outcomes of both rater groups: .920 for the Spanish raters and .971 for the Chinese raters.

### Spanish Translation: iTranslate Versus Human

We ranked the sentences based on their readability scores and presented the results with the easiest sentence first, followed by the medium, and put the most difficult sentence last (Table 3). Within the Fluency domain for iTranslate, the two relatively simple sentences (S2 and S3) had almost perfect fluency; however, the most difficult sentence (S1) had marginal fluency with several grammatical errors (Fluency=2). All the sentences translated by the Spanish human translator received excellent or perfect fluency scores (Fluency≥4). Within the Adequacy domain for iTranslate, S2 conveyed about 75% of the original information, S3 conveyed 100% of the original information, but S1 conveyed about half of the original information. All the sentences translated by the Spanish human translator conveyed most of the original information. Within the Meaning domain for iTranslate, S2 and S3 had almost the same meaning as the original, but S1 had practically the same meaning as the original. All the sentences translated by the Spanish human translator had almost the same meaning as the original. Within the Severity domain for iTranslate, S2 and S3 had almost no effect on patient care, but S1 had unclear effect on patient care. All the sentences translated by the Spanish human translator had (almost) no effect on patient care.

**Table 3:** Mean scores for Spanish iTranslate and the human Spanish translator.

Original sentences	Flesch-Kincaid grade level	iTranslate					Human			
		Fluency	Adequacy	Meaning	Severity	Fluency		Adequacy	Meaning	Severity
S2. What should they be?	0.0	4.67	4	4.33	4.67	4.67		3.67	3.67	5
S3. What actions should I take to reach these goals?	1.0	5	5	5	5	5		5	5	5
S1. What are my blood sugar, blood pressure, and cholesterol numbers?	7.1	2	3.33	3.33	3.67	4		4	4	4.67

### Chinese Translation: iTranslate Versus Human

As shown in Table 4, within the Fluency domain, all the sentences translated by both iTranslate and the Chinese human translator had excellent or perfect fluency. Within the Adequacy domain, all the sentences conveyed more than 75% to 100% of the original information. Within the Meaning domain, all the sentences had (almost) the same meaning as the original. Within the Severity domain, all the sentences had almost no effect on patient care.

**Table 4:** Mean scores for Chinese iTranslate and the human Chinese translator.

Original sentences	Flesch-Kincaid Grade Level	iTranslate					Human			
		Fluency	Adequacy	Meaning	Severity	Fluency		Adequacy	Meaning	Severity
S2. What should they be?	0.0	4	4.33	4.33	4.67	5		4.67	4.67	5
S3. What actions should I take to reach these goals?	1.0	4.67	4.67	4.67	5	5		4.67	4.67	5
S1. What are my blood sugar, blood pressure, and cholesterol numbers?	7.1	4.33	4.33	4.33	4.67	5		5	5	5

### Visually Comparing iTranslate and Human Versions

To better compare and capture the trends among sentences with regard to the quality scores on four domains, we created two graphs, presenting the findings of the easiest sentence (S2) first and the most difficult sentence (S1) last.

When sentences were translated from English to Spanish (Figure 2), for the easiest sentence (S2), there was a slight difference between iTranslate and the Spanish human translator, where iTranslate received slightly higher scores in the Adequacy (4 vs 3.67) and Meaning domains (4.33 vs 3.67), but slightly lower scores in the Severity domain (4.67 vs 5). There was no difference between iTranslate app and the Spanish human translator on S3 (the medium difficult sentence) in any of the four domains (5). For the most difficult sentence (S1), there was a slight difference between iTranslate and the Spanish human in the Adequacy and Meaning domains, where iTranslate received slightly lower scores (3.33 vs 4). We also noticed some gaps for S1 in the Fluency and Severity domains, where iTranslate received lower scores (2 vs 4 and 3.67 vs 4.67).

As shown in Figure 3, when sentences were translated from English to Chinese, there was almost no difference between the ratings of iTranslate app and the Chinese human translator on S3 (the medium difficult sentence) in any of the four domains. This funding is similar to the Spanish language. For the easiest sentence (S2) and the most difficult sentence (S1), there was a slight difference between iTranslate and the Chinese human translator, where iTranslate received slightly lower scores in all the four domains.

## Discussion

### Principal Findings

This pilot study compared the translation quality of iTranslate and professional human translators using three questions drawn from a diabetes patient education pamphlet. Materials were translated from English to Spanish and Chinese (Mandarin). We found iTranslate generally provided translation quality comparable to human translators on simple and medium difficulty sentences. The voice recognition feature and voice outputs employed by iTranslate produced text quality, clarity, and auditory richness (voice quality: native accent, tone, inflection, and delivery), which benefits individuals who cannot read in their native languages. However, iTranslate tends to make more errors when translating difficult sentences.

When translating the easiest sentence (ie, S2 “What should they be?”, Flesch-Kincaid Grade Level=0.0) from English to Spanish, the voice employed by iTranslate softened and deadened the [n] when pronouncing [deberían] so that the [n] almost sounds omitted. On the other hand, the Spanish human translator added the antecedent noun for the pronoun “they”. Therefore, even though the professional translator received slightly lower scores on the Adequacy and Meaning compared to iTranslate, the Spanish human translator received a slightly higher score on the Severity compared to iTranslate. One of the Spanish raters believed that S2 translated by iTranslate from English to Spanish had an unclear effect on patient care. When translating it from English to Chinese, iTranslate made no errors. Compared to the literal translation by iTranslate, the Chinese human interpreter added some extra information to clarify the word “they”, which translated the sentence into [正常值应该是多少？] (What should the normal range be?). Although iTranslate did not make any errors, the Chinese human version contained more specific and meaningful information. We believe this was the reason why iTranslate received slightly lower scores on all the four criteria compared to the Chinese human translator. Also, one of the Chinese raters believed that it had an unclear effect on patient care.

When translating the relatively easy sentence (ie, S3 “What actions should I take to reach these goals?”, Flesch-Kincaid Grade Level=1.0) from English to Spanish, the Spanish raters agreed that both versions had no effect on patient care even though the Spanish human interpreter substituted those [“esas”] for these [estas”]. Both iTranslate and the Spanish human interpreter received full scores on every criterion. When translating it from English to Chinese, iTranslate omitted the word “I” and translated this sentence into (“What actions should be taken to reach these goals?”). In comparison, the Chinese human interpreter substituted the phrase “take actions” into “do” and specified “these goals” into “normal numbers.” Therefore, the Chinese human interpreter translated S3 into [我应该怎么做来达到正常值？] (“What should I do to reach normal numbers?”). Neither iTranslate nor the human interpreter correctly translated S3 word for word; however, the general meaning of the original sentence has not been changed. Thus, all the raters agreed that S3 translated by either iTranslate or the Chinese human interpreter had no effect on patient care.

When translating the most difficult sentence (ie, S1 “What are my blood sugar, blood pressure, and cholesterol numbers?, Flesch-Kincaid Grade Level=7.1) from English to Spanish, iTranslate omitted the word “number.” Therefore, the Spanish evaluators believed it had marginal fluency with several grammatical errors, conveyed about half of the original information, had practically the same meaning as the original, and had an unclear effect on patient care or delays necessary care. On the other hand, the Spanish human interpreter did not make any errors when translating S1. When translating it from English to Chinese, iTranslate made the exactly same error as translating it from English to Spanish—it omitted the word “number” as well. Therefore, this sentence did not received full scores on Fluency, Adequacy, and Meaning, which led to one of the Chinese evaluators’ believing that such an error had an unclear effect on patient care. On the other hand, the Chinese human interpreter did not make any errors when translating S1. Interestingly, even though iTranslate made the exactly the same error on S1 for the Spanish and Chinese translations, the Spanish raters gave it lower scores on all the criteria than the Chinese raters did.

To minimize rater bias, we blinded the audio version of the translated question so that the raters could not identify the two audio versions (iTranslate and the human translations). However, rater bias might still exist because each rater had their interpretation of the evaluation rubric. Variability in the rating scores may result from bias (systematic error) or random error (unpredictable). For example, S1 translated by iTranslate from English to Spanish received lower scores on all the criteria than the same sentence translated by iTranslate from English to Chinese even though the Chinese and Spanish translations made the exact same error—omitting the head noun “numbers” in the nominal phrase “my blood sugar, blood pressure, and cholesterol numbers.” Usually there are number of ways to correctly translate a sentence; however, individuals might have different preferences on evaluating translation quality. Another example is Fluency. According to the rubric, 4 represents “excellent fluency” and 5 represents “perfect fluency.” We made no attempt to standardize the domain descriptors or train the raters; therefore, each evaluator might have a slightly different interpretation of “excellent” and “perfect.” Therefore, although we can make broad statements about the comparability and adequacy based on the scoring rubric of the human and machine translation in each language, we cannot conclude that iTranslate produces more accurate translations from English to Chinese than from English to Spanish.

Our findings appear to support iTranslate as producing competent, understandable translations for simple sentences. However, once the sentences get more complicated, iTranslate tends to make more errors. Previous studies documented high rate of errors in machine translations when translating written sentences. For instance, Sharif and Tse [28] identified half of the medicine labels translated by computer programs from English to Spanish as being incorrect. In another study, Khanna et al [22] found that Google Translate made more errors compared to human translators when translating patient education texts from English to Spanish. Chen et al [21] evaluated the accuracy of Google Translate when translating diabetes patient education materials from English to multiple languages (Spanish and Chinese). The authors reported that Google produced more accurate translations from English to Spanish than English to Chinese [21]. Turner et al [23] also reported a high error rate when Google translated health websites from English to Chinese. One explanation for the difference between our findings and the evidence noted above might result from sentence difficulty. Interestingly, our findings indicated that iTranslate was a relatively comparable tool when translating simple spoken sentences from English to Spanish and Chinese. We propose that the machine translation quality was comparable to the human translations only when the sentence was easier to understand due to the simplicity of the grammatical constructions. Hence, our results support the findings of Zeng-Treitler et al [24] who found that machine translation tools appear to be less likely to provide accurate translation for longer and more difficult sentences.

Guidelines are available for health professionals to work with human interpreters in clinical encounters [29-32]; however, to date and to the best of our knowledge, there are no recommendations or guidelines about using mobile translation apps. Randhawa et al [33] pointed out that machine translation devices have several potential benefits in clinical settings such as helping clarify patient histories, reviewing a clinical diagnosis, restating the recommended treatment plan, and encouraging patients to ask questions. Based on previous machine translation commentary studies [33,34] and our pilot data, we recommend that clinicians consider the following when interacting with LEP patients using mobile language translation apps as communication assistance tools: (1) use the mobile translation apps to supplement but not supplant human translators, and (2) provide information in clients’ and caregivers’ mother tongue about the mobile translation apps and how to use them, along with appropriate precautions.

### Limitations

This pilot study has several limitations. First, this study assessed the quality of the iTranslate mobile language translation app using only three spoken sentences from a diabetes patient education pamphlet. To compensate for the small number of sentence units, we investigated translations of these sentences from English into two languages (Spanish and Chinese). Second, we assessed only Spanish and Chinese translations so that the findings should not be applied to other languages. Future studies should investigate multiple machine translation tools with a larger sentence sample drawn from other public health materials as well as conversations from real clinical encounters. It is necessary to further investigate the relationship between machine translation error patterns and sentence complexity levels. Also, more studies should explore the app using experience from patients with LEP in various languages.

### Conclusions

To the best of our knowledge, this is the first study to evaluate and compare the quality of a mobile language translation app with a voice recognition feature and professional human translators. We found iTranslate could produce competent, understandable translations for simple sentences. However, once sentences became more complicated, iTranslate seemed to make more errors.

## Appendix

Multimedia Appendix 1 You are the heart of your family…take care of it.

## Abbreviations

ATA: American Translators Association
CDC: Centers for Disease Control and Prevention
LEP: limited English proficiency
