# Supplementary material for: Machine or Human? Evaluating the Quality of a Language Translation Mobile App for Diabetes Education Material
Source: JMIR Diabetes. 2017 Jun 29;2(1):e13. doi: 10.2196/diabetes.7446 (PMC6238892; doi:10.2196/diabetes.7446)
Supplement: Multimedia Appendix 1 [file diabetes_v2i1e13_app1.pdf]

You are the heart of your family ...take care of it.

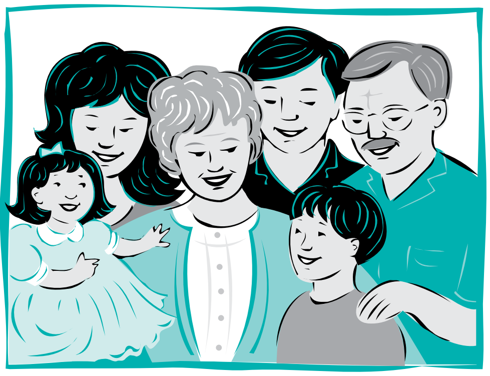

Having diabetes can lead to a heart attack or stroke — *but it doesn't have to.*

You can live longer for your family by taking care of your heart. Over time, high sugar levels in the blood can damage vital organs, such as your kidneys, your eyes, and your heart. High blood pressure is a serious disease that makes your heart work too hard. And bad cholesterol (LDL cholesterol) builds up and clogs your heart's arteries. Controlling these three medical problems leads to a longer and healthier life.

Ask your doctor these questions:

1. What are my blood sugar, blood pressure, and cholesterol numbers?
2. What should they be?
3. What actions should I take to reach these goals?

You can live longer for your family, improve your health, and reduce your risk of heart disease.

Take action now:

- Eat more fruits, vegetables, beans, and whole grains.
- Eat tasty foods that have less salt, saturated fat, and trans fat. Order a free recipe booklet and meal planner for making healthy meals by calling 1-800-860-8747.
- Get at least 30 minutes of physical activity on most days or every day. Physical activity helps you keep a healthy weight.
- Stop smoking—ask for help to quit. Call 1-800-QUIT-NOW (1-800-784-8669).
- Take medicines the way your doctor tells you.
- Ask your doctor about taking medicine to protect your heart, such as aspirin or a statin.

Ask your family and friends to help you take care of your heart and your diabetes.

Use this chart to keep track of your A1C, blood pressure, and cholesterol numbers when you visit your doctor. Work with your doctor, friends, and family to reach your goals.

GOAL: TAKE CARE OF YOUR HEART

Talk to your doctor about what your goals should be.

1. A1C

The A1C test—short for hemoglobin A1C—measures your average blood sugar over the last three months.

Suggested A1C Goal for Many People: **Below 7**

Test at least twice a year

Date ______________________________

Result ______________________________

2. BLOOD PRESSURE

High blood pressure makes your heart work too hard.

Suggested Blood Pressure Goal for Most People: **Below 140/90** unless your doctor helps you set a different goal.

Test at every visit

Date ______________________________

Result ______________________________

3. CHOLESTEROL

Bad cholesterol, or LDL cholesterol, builds up and clogs your heart arteries.

Suggested Goal: **Ask what your cholesterol numbers should be.**

Test at least once a year

Date ______________________________

Result ______________________________

National Institutes of Health

1-800-860-8747 • TTY: 1-866-569-1162
www.ndep.nih.gov

This information is not copyrighted. The NIDDK encourages people to share this content freely.

July 2014 NIH Pub. No. 14-50585 NDEP-58

NDEP National Diabetes Education Program

A program of the National Institutes of Health and the Centers for Disease Control and Prevention
